# Multiple layers of intratumor heterogeneity: clues to clonal evolution of non-small cell lung cancer

**DOI:** 10.18632/oncotarget.26708

**Published:** 2019-02-22

**Authors:** Steffen Dietz, Daniel Kazdal, Holger Sültmann

**Affiliations:** Division of Cancer Genome Research, German Cancer Research Center (DKFZ) and National Center for Tumor Diseases (NCT), Heidelberg, Germany; Translational Lung Research Center (TLRC) Heidelberg, German Center for Lung Research (DZL), Heidelberg, Germany; German Cancer Consortium (DKTK), Heidelberg, Germany

**Keywords:** tumor heterogeneity, clonal evolution, lung cancer, copy numbers, epigenomics

Lung cancer is among the most prevalent cancers and a leading cause of malignancy-related death [1]. For a long time, the most frequent subtype, non-small cell lung cancer (NSCLC), has been diagnosed at the cellular level using histopathological differentiation between entities, in particular adenocarcinoma (ADC) and squamous cell carcinoma (SCC). Later, ADC was further subdivided into five distinct morphological growth patterns (lepidic, acinar, papillary, micropapillary, and solid) with prognostic relevance [2]. However, precise diagnosis remains challenging due to extensive intratumor heterogeneity (ITH) and the co-occurrence of several growth patterns within individual tumors [2].

**Figure 1:**
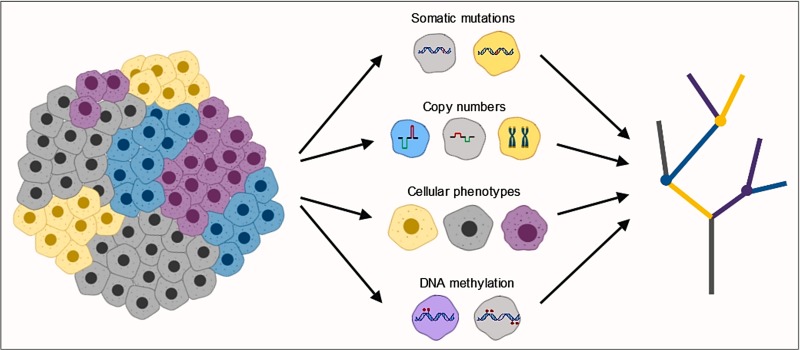
Subclonal diversity within a tumor leads to spatial and temporal intratumor heterogeneity Tumor subclones differ in cellular phenotypes, somatic mutations, copy number variations as well as DNA methylation, all of which contribute to this heterogeneity. Integrative, multi-regional analysis may help to understand the causes and consequences of intratumor heterogeneity.

This morphological ITH goes along with multiple molecular alterations within primary and metastatic lesions of the same individual: Whole-exome sequencing of spatially separated tumor regions demonstrated extensive genetic and genomic ITH (Figure 1) [3]. Approximately 30% of somatic mutations and 50% of copy number variations (CNVs) were identified as subclonal and present in at least one, but not all, tumor regions. Furthermore, multiregion sequencing provided evidence for distinct mutational processes, genome doubling, and increased chromosomal instability as factors contributing to molecular ITH. Multiple subclonal CNVs were associated with disease recurrence or survival, thereby linking ITH to clinical outcome [3]. Phylogenetic analysis of the molecular data indicated that ITH is a driver of tumor evolution and that branched NSCLC evolution is almost pervasive [3, 4].

Despite this considerable ITH, only few studies performed an integrative analysis of molecular and morphological markers in NSCLC. To comprehensively compare the spatial distribution of *EGFR* and *KRAS* alterations with tumor cell content and the predominant morphological growth pattern, we recently analyzed central sections of 19 ADCs subdivided into 467 5×5 mm segments. We demonstrated that *EGFR* and *KRAS* driver mutations were present in all malignant segments, suggesting a clonal origin. Despite high levels of inter-and intratumor heterogeneity, we found a significant correlation between variant allele frequencies (VAFs) and morphological growth patterns, but not with tumor sizes [5]. VAFs were highest in segments with a predominant solid pattern.

In addition to genomic ITH we addressed somatic mutations of the mitochondrial genome (mtDNA), which were either ubiquitously distributed throughout a tumor section or restricted to specific regions [6]. Spatial and histological mapping of these mutations enabled the identification of subclonal structures and phylogenetic relations within a tumor. ADC with more than two ubiquitous mtDNA mutations were associated with shorter disease-free survival, suggesting a prognostic relevance of somatic mtDNA mutations.

To analyze tumor heterogeneity on the epigenomic level, we recently used a similar segmentation approach [7] and determined genome-wide DNA methylation and copy number profiles in spatially distinct regions of 27 primary tumor segments, matched normal lung tissues, and six lymph node metastases from the seven ADC patients. We found notable spatial variation of DNA methylation levels within and between cases. Regional ITH was not only present between segments with distinct morphological growth patterns, but also between spatially separated segments of the same growth pattern. Samples with the same morphology were not necessarily more similar to one another than to those of other cases. These findings were not only obtained by unsupervised hierarchical clustering CpG sites with the highest methylation variability, but also when only enhancer and promoter CpG sites were used. Notably, this intratumor methylation heterogeneity might have a direct effect on gene expression and lead to regionally differing expression patterns throughout the tumors. To put our data in a broader context, we performed unsupervised clustering of 12,601 CpG sites in promoter regions using the 33 tumor segments and the corresponding sites in 369 cases (366 tumor and 38 normal samples) from the TCGA lung ADC cohort [8]. We found that spatially separated regions from one individual were represented in distant clusters. Thus, our small sample set demonstrated that the extensive intratumor DNA methylation heterogeneity of ADCs is independent of the various morphological growth patterns.

To infer the phylogenetic relationships between the segments, we calculated distance matrices based on DNA methylation and CNVs. In line with a previous publication [3], methylation-based phylogenies demonstrated a branched evolution of ADCs with extensive subclonal diversification between growth patterns in the primary tumors. These findings suggest that a particular cellular phenotype can emerge independently in distinct tumor regions at different time points during tumor evolution. Although CNV profiles were generally more similar within than between tumors, we identified subclonal copy number gains and losses encompassing potential tumor driver and suppressor genes. Combined morphology, DNA methylation, and CNV data reinforced the notion that a given biopsy from a single tumor region only reflects a fraction of the tumor's phenotypic and molecular profile.

Increasing evidence suggests a crucial role of ITH as a driver of tumor progression and therapy failure. Hence, ITH poses a major challenge for the treatment and prognosis of patients. However, only little is known about epigenomic ITH to date. Our results suggest that extensive inter-as well as intra-tumor variation shape the molecular and phenotypic heterogeneity of ADCs. The challenge of future research will be to understand the interplay between these multiple genomic and epigenomic layers (Figure 1). While a systematic analysis of larger cohorts is warranted to validate these findings, the concept of NSCLC as an assembly of tumor clones evolving and adapting in space and time is consolidating.

To overcome the limitations of single tissue biopsies and to avoid repeated multiregional tissue sampling, novel approaches are required. To this end, tumor DNA circulating in the blood might depict tumor heterogeneity and clonal evolution under therapy more comprehensively [9, 10]. However, to which extent such liquid biopsy approaches provide useful information for the clinician remains to be explored.
